# An Integrated Literature Review of Time-on-Task Effects With a Pragmatic Framework for Understanding and Improving Decision-Making in Multidisciplinary Oncology Team Meetings

**DOI:** 10.3389/fpsyg.2019.01245

**Published:** 2019-07-09

**Authors:** Tayana Soukup, Benjamin W. Lamb, Matthias Weigl, James S. A. Green, Nick Sevdalis

**Affiliations:** ^1^Centre for Implementation Science, Health Service and Population Research Department, Institute of Psychiatry, Psychology and Neuroscience, King’s College London, London, United Kingdom; ^2^Department of Urology, Cambridge University Healthcare NHS Foundation Trust, Cambridge, United Kingdom; ^3^Institute and Outpatient Clinic for Occupational, Social, and Environmental Medicine, University Hospital, Ludwig-Maximilians-University Munich, Munich, Germany; ^4^Whipps Cross University Hospital, Barts Health NHS Trust, London, United Kingdom

**Keywords:** decision making, cognitive fatigue, mental fatigue, self-control, multidisciplinary oncology team meetings, tumor boards, intervention, cognitive strategies

## Abstract

Multidisciplinary oncology team meetings (MDMs) or tumor boards, like other MDMs in healthcare, facilitate the incorporation of diverse clinical expertise into treatment planning for patients. Decision-making (DM) in relation to treatment planning in MDMs is carried out repeatedly until all patients put forward for discussion have been reviewed. Despite continuing financial pressure and staff shortages, the workload of cancer MDMs, and therefore meeting duration continue to increase (up to 5 h) with patients often receiving less than 2 min of team input. This begs the question as to whether the current set-up is conducive to achieve optimal DM, which these multi-specialty teams were set out to achieve in the first place. Much of what it is known, however, about the effects of prolonged cognitive activity comes from various subfields of science, leaving a gap in applied knowledge relating to complex healthcare environments. The objective of this review was thus to synthesize theory, evidence and clinical practice in order to bring the current understanding of prolonged, repeated DM into the context of cancer MDMs. We explore *how* and *why* time spent on a task affects performance in such settings, and *what* strategies can be employed by cancer teams to counteract negative effects and improve quality and safety. In the process, we propose a pragmatic framework of repeated DM that encompasses the strength, the process and the cost-benefit models of self-control as applied to real-world contexts of cancer MDMs. We also highlight promising research avenues for closing the research-to-practice gap. Theoretical and empirical evidence reviewed in this paper suggests that over prolonged time spent on a task, repeated DM is cognitively taxing, leading to performance detriments. This deterioration is associated with various cognitive-behavioral pitfalls, including decreased attentional capacity and reduced ability to effectively evaluate choices, as well as less analytical DM and increased reliance on heuristics. As a short to medium term improvement for ensuring safety, consistently high quality of care for all patients, and the clinician wellbeing, future research and interventions in cancer MDMs should address time-on-task effects with a combination of evidence-based cognitive strategies. We propose in this review multiple measures that range from food intake, short breaks, rewards, and mental exercises. As a long term imperative, however, capacity within cancer services needs to be reviewed as well as how best to plan workforce development and service delivery models to achieve population coverage whilst maintaining safety and quality of care. Hence the performance detriments that arise in healthcare workers as a result of the intensity (time spent on a task) and complexity of the workload require not only more research, but also wider regulatory focus and recognition.

## Multidisciplinary Oncology Teams

As an important part of cancer care services, multidisciplinary teams (MDTs), like other MDTs in healthcare, facilitate integration of a wide range of core clinical disciplines, including oncologists, radiologists, pathologists, surgeons, physicians and specialist cancer nurses. In many healthcare systems globally, e.g., in the United Kingdom, the needs of patients with suspected or confirmed cancer are addressed at weekly MDT meetings (MDMs) or tumor boards. Cancer MDMs lead to the formulation of expert informed treatment recommendations, and as such, they are arguably an invaluable part of the cancer care pathway. There is a general understanding that such a multidisciplinary approach is better placed to adequately address complexity of cancer care (Department of Health, 2004; National Cancer Action Team, 2010), with wider scientific evidence lending support to such approach (Kugler et al., 2012). Due to an increasingly complex and specialized care, multidisciplinary teams have become integral to effective service delivery and quality, while effective team working presents an important aspect of non-technical skills that promotes patient safety (Kohn et al., 2000; Cosby and Croskerry, 2004; Reader et al., 2006, 2011; Manser, 2008; Vincent, 2010; Kugler et al., 2012; Weller et al., 2014; Francis, 2015; Gluyas, 2015).

The currently outstanding question, therefore, may not be so much concerning the *why* component, i.e., why convene MDMs, but the *how* – i.e., how to make them more effective and efficient. This is an important question to address since converging evidence shows that although cancer survival rates may be improved by MDMs (Kesson et al., 2012), performance in MDMs is variable with commonly occurring patterns of unequal specialist participation and suboptimal information sharing (Fleissig et al., 2006; Tattersall, 2006; Lamb et al., 2011, 2013a, Lingard et al., 2011; Arora et al., 2012; Jalil et al., 2013; Seretis et al., 2014; Soukup et al., 2016a,b).

**FIGURE 1:**
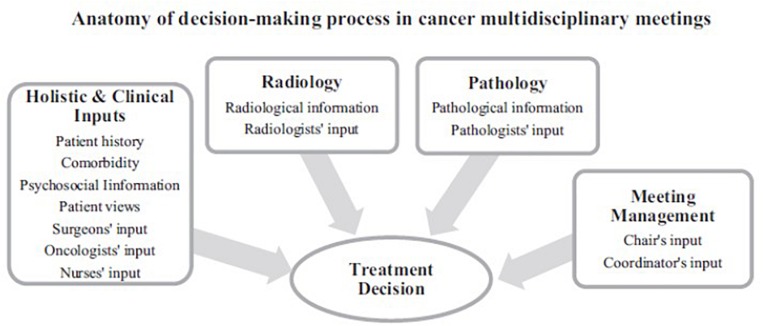
Diagram depicting the anatomy of decision-making in cancer multidisciplinary team meetings.

To answer the question of how to improve the efficiency and effectiveness of MDMs we need to better understand the underlying intricacies of DM in MDM and the reasons for these variations. The decision-making (DM) process in MDMs is complex (Figure 1). For each patient on the meeting agenda, the MDT needs to consider a range of clinical information in order to formulate a treatment recommendation, such as patient’s medical history, radiological information (incl. images and report), histopathological information (incl. images and report), patient comorbidities, psychosocial aspects, as well as patient views and preferences on the treatment options. For each patient on the meeting agenda, this information needs to be critically discussed by all core members of the team. Typically, these include surgeons, radiologists, histopathologists, oncologists, specialist cancer nurses and physicians. Following the discussion, the core members need to reach a consensus on the best treatment option for every patient on the meeting agenda until all patients put forward for the MDM have received a decision (Fleissig et al., 2006; Tattersall, 2006; Lamb et al., 2011, 2013a, Lingard et al., 2011; Arora et al., 2012; Jalil et al., 2013; Seretis et al., 2014; Soukup et al., 2016a,b). Figure 1 demonstrates this process. Moreover, the evidence shows that all aspects of aforementioned clinical information as well as contribution to discussion by all core disciplines are critical for the team to reach and subsequently implement the decision (Lamb et al., 2013b; Soukup et al., 2016a,b).

**FIGURE 2:**
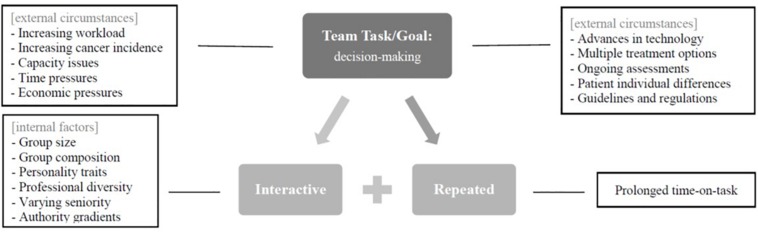
Diagram depicting the fundamental nature of decision-making in cancer meetings that is interactive and repeated together with the additional layers of complexity emanating from within and outside individual teams, namely, increased workload, time pressure, multiple treatment options and patient individual differences.

The very nature of DM in MDM therefore requires teams to seek and process information, both cognitively and interactively (with other members) on a consecutive basis for a prolonged period, and in the face of multiple concurrent challenges (Figure 2), such as:

• *Increasing workload and time pressures* as a result of rising cancer incidence (Mistry et al., 2011; World Health Organization [WHO], 2014), financial pressures (NHS England, 2014; World Health Organization [WHO], 2014) and staff shortages (NHS Improvement, 2016);
• *Multiple treatment options* as a result of continuous advances in cancer treatments and preventions as well as ongoing cancer trials, which the MDT should consider;
• *Patient individual differences and circumstances*; and
• Patient preferences and choice of treatment.

Consequently, the duration of MDMs is increasing over time, such that prolonged, repeated DM has become a norm for many teams, particularly for the more common cancers (e.g., gynecological, breast, colorectal and urological). In many instances, meetings may last up to 5 h with each patient receiving less than 2 min of team input (Cancer Research UK, 2017).

*This begs the question of whether the current arrangement is conducive to effective and optimal DM, which these multi-specialty teams were set out to achieve in the first place.* Here we propose that such a complex and intense cognitive workload, coupled with prolonged and interactive time spent on repeated formulation of treatment recommendations, leads to detriments in performance and outcomes (i.e., time-on-task). While management of excessive working hours and sleep deprivation amongst healthcare workers is widely recognized as a phenomenon linked to adverse events and reduced patient safety, the scientific efforts in healthcare lack focus on the intensity (time spent on a task, or time-on-task) and complexity of the workload during the working hours (Barger et al., 2006; World Health Organization [WHO], 2009; The Joint Commission, 2011). This is despite healthcare being fraught with examples of intense cognitive work (Reid et al., 2005; Reader et al., 2006, 2011). Hence little is known about how this impacts on clinical performance (Flindall et al., 2016). Two studies have so far explored this issue demonstrating positive association between the cases at the start of the meeting and the quality of DM in urology MDMs (Lamb et al., 2013a) and more recently, in breast cancer MDMs (Soukup et al., 2019a). In other clinical settings, it was shown that the quality of endoscopy performance (Flindall et al., 2016) and clinical handovers (Flindall et al., 2016) declines with successive/repeated procedures.

It remains unresolved how the quality of DM in cancer MDMs is affected by repeated cognitive efforts (i.e., time-on-task). This is important to address, since both improvements in quality and cost effectiveness can be achieved through gains in efficiency (Department of Health, 2010; Hurst and Williams, 2012). A healthcare system is considered efficient if it avoids waste, not only of the equipment and supplies, but also of ideas and energy (Reid et al., 2005). Placing the current scientific knowledge of prolonged, repeated DM into the context of cancer MDMs is therefore a critical first step in (a) defining and understanding the problem, and (b) proposing strategies and interventions to maintain the appropriate levels of cognitive load during meetings. Eventually, this preserves team’s efforts required for consecutive DM in the face of concurrent challenges, thus ensuring both, effectiveness and efficiency.

In the light of recent systematic reviews and meta analyses on the topic of time-on-task (Hagger et al., 2010; Carter et al., 2015; Tuk et al., 2015), none of which addresses cancer MDMs, we present a comprehensive synthesis of the current scientific understanding, and also discuss its contextual clinical relevance and future directions. We further propose a pragmatic framework of repeated DM for the complex interactive setting of MDMs. Focused on strategies to prevent detriments in performance within current resources and in the face of concurrent challenges (Figure 2), this review attempts to address three specific questions in relation to cancer MDMs:

(1) Why does prolonged time-on-task affect task performance (section Theories and Models of Prolonged Time-on-Task)?
(2) How does prolonged time-on-task affect task performance (section Cognitive-Behavioral Pitfalls associated with Time-on-Task)?
(3) What strategies exist as countermeasures of time-on-task effects (section Cognitive-Behavioral Strategies as Countermeasures of Time-on-Task Effects)?

## Search Strategy and Selection Criteria

The literature for this review was obtained via the electronic searches on PubMed, Scopus, Web of Science, OVID Medline, and OVID PsycINFO including PsyArticles. The search in relation to the intervention studies presented in Table 1 was performed for the period between 1990 and 2015 (last search in relation to Table 1 was on 14 August 2015). Articles were also identified through the searches of 3 recent meta analyses concerned with the effects of time-on-task on task performance (i.e., Hagger et al., 2010; Carter et al., 2015; Tuk et al., 2015). The remaining articles included in this review were generated on the basis of originality and relevance to cancer team DM and the broad scope of this literature review. We considered papers published in English language that included non-disordered human population group of adult age, which encompassed original research articles, as well as evidence reviews. Personal commentaries and studies involving animal subjects were not considered.

**TABLE 1:** Overview of cognitive strategies used in the literature as intervention to improve performance decrements from prolonged cognitive activity.

**Intervention**		**Authors**	**Study design**	***N***	**Self-control task**	**Finding**
Break and time of day	5 min break with stretching at 2 separate intervals	Galinsky et al.,2007	Field study (data-entry workers).	51	Data entry.	Improvement in speed without loss of productivity.
	Five short breaks spaced hourly over 6 h flight	Neri et al.,2002	Field study (a simulated flight study with a crew).	14	Objective vigilance. Subjective vigilance. Physiological measure (EEG/EOG).	No improvement. Improvement. Improvement in the rate of unintended sleep episodes, theta-band activity (associated with working memory), and slow eye movement.
	10 min break with relaxing music	Tyler and Burns,2008	Experimental.	41	Stroop task.	Improvement in speed and accuracy.
	5 min of mindfulness mediation	Friese et al.,2012	Experimental.	66	Test of attention.	Improvement.
	1st half of the day	Kouchaki and Smith,2014	Experimental.	337	Moral decision-making task.	Improvement.
Food and drinks	Ingesting glucose (sweetened drink)	McMahon and Scheel,2010	Experimental.	50	Probability-learning task.	Improvement.
		Masicampo and Baumeister,2008	Experimental.	121	Consumer decision task.	Improvement (with decrease in reliance on heuristic-based automatic reasoning).
		Owen et al.,2012	Experimental (double-blind, placebo controlled, six-way crossover study).	30	Word presentation. Immediate word recall. Serial threes (arithmetic). Serial sevens (arithmetic). Stroop task. Simple reaction time. Choice reaction time. Alertness (subjective report) Contentedness (subjective) Calmness (subjective)	Improvement. Improvement. Improvement. Improvement. No improvement. No improvement. Improvement. Improvement. No improvement. Improvement.
		Kennedy and Scholey,2000	Experimental (double-blind, placebo controlled, balanced crossover).	20	Serial sevens (arithmetic). Serial threes (arithmetic). Word retrieval.	Improvement. Improvement. Improvement.
		Scholey et al.,2001	Experimental (placebo controlled double-blind balanced crossover).	20	Serial Sevens (arithmetic). Word Fluency task. Word Recall task.	Improvement. Improvement. No improvement.
		Reay et al.,2006	Experimental (placebo controlled double-blind balanced crossover).	27	Serial Threes (arithmetic). Serial Sevens (arithmetic). Rapid visual information processing task. Participant report of mental fatigue (visual analog scale).	Improvement in total number of subtractions in both tasks. Improvement in accuracy. Improvement in subjective reports of fatigue.
	Caffeine (3 mg/kg body weight)	Tieges et al.,2004	Experimental (double-blind, placebo controlled, cross-over design).	15	Alternating runs task. Physiological measure (EEG)	Improvement in error rate and accuracy Increased ERN amplitude in ACC.
	Ingesting caffeine and glucose	Kennedy and Scholey,2004	Experimental (double-blind, placebo controlled, cross-over).	56	Serial Threes. Serial Sevens. Rapid Visual Information Processing Rating of mental fatigue.	No improvement. Improvement with caffeine only. Improvement with caffeine-glucose. Improvement with caffeine-glucose.
Reawards and motivation	Rinsing mouth with glucose (as a motivational strategy)	Hagger and Chatzisarantis,2013	Experimental (includes 5 replication studies).	183	Handgrip persistence. Figure-tracing. Problem-solving. Stroop. Counting.	Improvement. Improvement. Improvement. Improvement. Improvement.
		Molden et al.,2012	Experimental.	31	Stroop task.	Improvement.
		Sanders et al.,2012	Experimental (replication of Molden et al., 2012).	51	Stroop task.	Improvement.
	Monetary reward	Krebs et al.,2010	Experimental.	36	Stroop task.	Improvement.
	Monetary reward	Goto and Kusumi,2013	Experimental.	46	Handgrip persistence task.	Improvement.
	Altruistic motivation and positive belief about outcome	Muraven and Slessareva,2003	Experimental.	227	Puzzles and frustrating game.	Improvement in goal persistence in both tasks.
	Priming positive mood and rewards (e.g., by watching a comedy video, or receiving a surprise gift)	Yvo et al.,2014	Experimental.	160	Weight-lift persistence task.	Improvement in goal persistence.
		Tice et al.,2007	Experimental.	204	Tasting beverage. Frustrating Solvable task. Frustrating Unsolvable task. Handgrip persistence task.	Improvements in goal persistence.
Cognitive exercises	Mental imagery of a restorative activity, i.e., perspective-taking	Egan et al.,2012	Experimental.	190	Frustrating Unsolvable task. Handgrip persistence task.	Improvement in goal persistence in all tasks.
	Monitoring task performance against a set standard or criteria	Wan and Sternthal,2008	Experimental.	181	Frustrating Solvable task. Frustrating Unsolvable task.	Improvement in all tasks.
	Practicing logical reasoning with daily mental exercises using paper and pen workbook	Bertrams and Schmeichel,2013	Experimental.	49	Problem solving task (anagram).	Improvement anagram performance.
	Practicing self-control with daily internet-based training application based on Stroop task paradigm	Cranwell et al.,2014	Experimental.	62	Counting task. Handgrip persistence task.	Improvement in persistence and speed.

## Theories and Models OF Prolonged Time-on-Task (The Why)

Unpacking the existing scientific understanding, theories and models can help us gauge the significance of prolonged cognitive activity for task performance, and its relevance to cancer MDTs. See Appendix for the definitions of key concepts introduced in this section.

### Decision-Making and Self-Control

As a higher-order cognitive ability, DM is a goal-directed behavior concerned with making predictions and selecting the most viable option among a set of alternatives, in the face of uncertainties about the consequences of these options (Garavan et al., 2002). In the context of cancer MDMs, DM involves weighing up and comparing the predicted consequences of treatment options, while considering all available patient information (Mitchell, 2013). As such, DM requires self-regulation and executive control. The prefrontal cortex plays an important role in this process as it sends signals to other brain regions to inhibit irrelevant activity and promote task-relevant processing (Garavan et al., 2002; Hare et al., 2009; Figner et al., 2010; Wang, 2008, 2012). Successful self-control is therefore pivotal to effective DM as it allows one to override the initial response, behavior and action, through a stable attentional engagement and employment of multiple cognitive domains in order to execute a goal (Garavan et al., 2002; Hare et al., 2009; Figner et al., 2010; Wang, 2012). In MDMs, these goals are treatment recommendations (see Figures 1, 2).

### Time-on-Task Effects

Self-control and executive function are implicated in various elements of human behavior including DM, choice and volition, attention, emotion, cognitive impulse control, consumption, health behaviors, food choices, driving and sports, and has therefore been studied in many subfields of science – from psychology, neuroscience and behavioral economics to organizational and consumer behavior (Hagger et al., 2010; Carter et al., 2015; Tuk et al., 2015). There is a general understanding that self-regulation and executive control can be exhausted as a result of previous efforts (i.e., time-on-task). Such prolonged periods of cognitive activity lead to deterioration in information processing functions, increases of undesirable behavior and performance detriments. These have been studied on various self-control tasks (e.g., anagrams, puzzles, Stroop paradigm, hand grip, food consumptions, working memory, and standardized tests). Evidence suggests that in the sequential task paradigms that require self- and executive control at Time 1, a negative impact on the self- and executive control can be observed at Time 2 (Hagger et al., 2010; Carter et al., 2015; Tuk et al., 2015). Three recent meta-analyses corroborate significant effects of time-on-task on performance, although with varying effect sizes and statistical heterogeneity (43% for standardized tests and up to 88% for food consumption; Carter et al., 2015), because of between study variations and different outcome tasks used across studies:

• Carter et al. (Carter et al., 2015): *N* = 18, ***d* = 0.24** for possible anagrams to *d* = **0.79** for impossible puzzles;
• Hagger et al. (2010): *N* = 83, ***d* = 0.62**; and
• Tuk et al. (2015): *N* = 18, ***d* = 0.17**.

These meta-analyses suggest that the evidence for deterioration in task performance over time spent on a task is variable and the strength of the effects is not always consistent; note the discrepancy in effect size between Hagger et al. (2010) and Tuk et al. (2015). Challenges in testing and replicating these effects have therefore been documented, and recently also discussed in some detail in a review by Lurquin and Miyake (2017). However, most findings to date are based on laboratory studies with genuine lack of field research exploring the phenomenon; the exceptions are Harewood et al. (2009), Lamb et al. (2013a) and Soukup et al. (2019a) in clinical settings, and Danziger et al. (2012) in courtroom. The knowledge base around how such effects fair in complex cognitively demanding clinical settings is yet to be further built and developed.

Hence, it remains to be explored how cancer MDMs may also be affected by time-on-task effects with the MDT members engaging in DM in a sequential manner, case after case, for a prolonged period, i.e., ranging approximately from 60 to 320 min. This range is caused due to the variations in services across teams and hospitals, and the prevalence of individual cancers. For instance, for common cancers, such is breast cancer, the MDT is likely to have longer meetings than for the less common cancers, such as head and neck (Lamb et al., 2013a; Soukup et al., 2016a; Cancer Research UK, 2017). Such consecutive cognitive efforts could lead to self-control exhaustion, where mere act of making repeated treatment recommendations depletes the very same neurocognitive functions (i.e., executive functions and self-control) that support the DM itself, thus making subsequent self-control and decisions more challenging and error-prone (Garavan et al., 2002; Wang, 2008, 2012; Hare et al., 2009; Figner et al., 2010; Hagger et al., 2010; Danziger et al., 2012; Mitchell, 2013; Carter et al., 2015; Tuk et al., 2015; Ceschi et al., 2017; Lurquin and Miyake, 2017). Correspondingly, the preliminary evidence on DM in cancer MDMs suggests a negative impact (Lamb et al., 2013a; Soukup et al., 2019a).

While evidence on detrimental effects of prolonged time-on-task has been documented over the recent decades (although with varying effect sizes; Hagger et al., 2010; Carter et al., 2015; Tuk et al., 2015), different theories and models have emerged to explain it. To provide a more in depth understanding of behavior in a complex setting of cancer MDMs, we propose a pragmatic framework that integrates the current theories and models. In the process, we unpack the current theories and models, highlighting their shortfalls in relation to their applicability to cancer MDMs while building an understanding of how together they can complement one another when it comes to explaining the phenomenon in such complex non-experimental settings.

### The Strength Model of Self-Control

*The strength model* of self-control (aka ego depletion) suggests that detrimental effect arises because of cognitive control and information processing being limited resources, exhaustive in short term (Baumeister, 2002, 2003, 2014; Schmeichel et al., 2003). The analogy is often drawn with physical activity after a period of sustained exertion. It is thus argued that self-control can also become depleted when cognitive demands are made consecutively. Since one may not necessarily be consciously aware of being cognitively fatigued, susceptibility to cognitive errors is high, as described in the proceeding section on cognitive-behavioral pitfalls (Baumeister, 2002; Schmeichel et al., 2003).

### The Process Model of Self-Control

In contrast, *the process model* describes that the detrimental effect is a consequence of a change in motivation and attention from “have-to” tasks – tasks that are externally mandated (e.g., reviewing clinical details for patients and formulating recommendations) – requiring self-control at Time 1 to “want to” tasks – tasks that are enjoyable, less laborious and intrinsically rewarding, e.g., resting and food consumption – at Time 2 (Kurzban et al., 2013; Inzlicht et al., 2014; Inzlicht and Schmeichel, 2012).

### The Cost-Benefit Model of Self-Control

On a physiological level, *the cost-benefit model* with a cost being the energy required for subjective effort at self- and executive control, offers a plausible complementary explanation that brings closer *the strength* and *process models* since some level of control of one’s cognitive resources and responses is needed, and is fundamental to all goal-directed behaviors, such is DM (Boksem and Tops, 2008; Kurzban et al., 2013; Wagner, 2013). Energy is the most valuable resource for an organism, and its efficient use is therefore important (Boksem and Tops, 2008); as such the energy exerted for self- and executive control can be seen as a limited resource (as per *the strength model*). The brain is an organ with high energy demand – i.e., although it weighs only about 2% of body weight, it consumes about 20% of energy used by the entire body (Roth and Dicke, 2005). When depleted, however, the brain does not stop working, it becomes less efficient at appropriately evaluating the potential bio-energetic costs and benefits of available options. Consequently, the brain responds more strongly to the immediate rewards, i.e., immediate high benefit and low cost, paying less attention to the available information and the long-term consequences of those choices (Kool et al., 2010; Wagner, 2013). The immediate benefits therefore start to compete with the set goal-directed intentions, which may lead to shifts in attention and motivation from “have to” to “want to” tasks, i.e., those that are more enjoyable and intrinsically rewarding (as per *the process model*).

What is more, it has been argued that, in line with one’s mental representation of the costs and benefits associated with the task at hand, fatigue acts as an adaptive signal for the cognition to change current behavioral strategy and opt for lower energy and effort alternatives and higher immediate rewards (Boksem and Tops, 2008; Kurzban et al., 2013). This proposition arguably integrates the *strength* and *process models* and is in line with a time-honored principle of human behavior, namely *the law of less work or principle of least effort* (Kool et al., 2010).**** It is also in line with the *energetic evolutionary model of the human neurocognitive architecture* (LaCerra and Bingham, 1998; LaCerra, 2003) where it is argued that the brain in its effort to maintain ordered biological state prioritizes the most energetically optimal alternatives. Hence, when it comes to DM, it is less energy taxing to maintain status quo and not to engage cognitive resources. Energetic input (e.g., glucose), or energetic conservation (e.g., rest) must exceed, on average, the energetic output (e.g., self-regulation). If it does not, the brain becomes less efficient with a reduction in task performance, since the bio-energetic costs are too high (LaCerra and Bingham, 1998; LaCerra, 2003; Roth and Dicke, 2005; Boksem and Tops, 2008; Friston, 2010; Kool et al., 2010; Wagner, 2013). Addressing the immediate, short-term needs such as rest and food, for instance, may become an important step in effectively accomplishing a task (Boksem and Tops, 2008).

### The Pragmatic Framework of Repeated Decision-Making for Oncology Team Meetings

**FIGURE 3:**
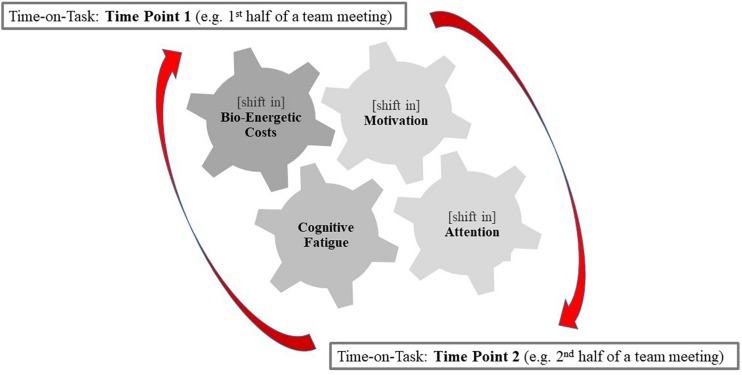
Diagram depicting a pragmatic framework of repeated decision-making in multidisciplinary oncology meetings.

To explain the complex and multifactorial nature of cognitively demanding organizational settings such are MDMs, a more integrative approach is needed. We therefore propose a pragmatic framework of repeated DM presented in Figure 3 that builds upon the process model in Inzlicht et al. (2014) by integrating it with the cost-benefit and strength models. For instance, in many controlled experimental studies presented in this review, participants are tested for a fraction of the time of an actual MDM and in controlled environments that encompass less complex interactions: e.g., short tasks for as long as 12 min only, except for 2 h in Lorist et al. (2005) and 3 h in Lorist (2008), in comparison to duration and frequency of DM in MDMs that can in some instances reach up to 5 h (Cancer Research UK, 2017). Hence detriments in performance over time-on-task in cancer MDMs arise from a simultaneous combination of factors (i.e., fatigue, task complexity, information load, as well as motivation and attention) due to the cognitive control being stretched beyond its limits. This encompasses experiences of fatigue (*the strength model*), shifts in attention and motivation from “have to” to “want to” tasks (*the process model*), as well as the shifts toward immediate energetically high benefit and low cost (*the cost-benefit model/the energetic evolutionary model/the law of least effort*) as demonstrated in Figure 3. For the exceptional circumstances of MDMs with up to 5 h spent on repeated and interactive formulation of treatment decisions, it may therefore be challenging to pull apart the above-mentioned models due to potential interaction effects – a premise that is yet to be directly tested in this context. See Figure 3 for a graphical representation of this argument and a pragmatic framework of repeated DM that we propose.

### Occupational Fatigue

In occupational health and safety sciences, there is a large and longstanding research on work-related fatigue. It is frequently defined as a state “of diffuse sensation of weariness [..] that is distressing if we cannot allow ourselves to relax” (Åhsberg, 2000). Mental fatigue arises in the course of working on mentally demanding tasks that need to be performed over time with the results of decrements in cognitive resources (e.g., alertness, reasoning, vigilance). Occupational fatigue is a dynamic and multifactorial phenomenon influenced by (1) *task*, (2) *process, and* (3) *person-related factors* with considerable intra-personal variability (Dawson et al., 2011, 2012, 2017; de Bloom et al., 2015; Fan and Smith, 2017; Bakker and Demerouti, 2018; Caldwell et al., 2019). Among (1) *task characteristics*, several risk factors have been identified such as monotony (boredom), repetitive tasks, limited variability (i.e., skill variety, low discretion) as well as workload (Gander et al., 2011; Caldwell et al., 2019). Task duration has a critical influence, yet, its effect is dependent on the amount of mental effort; such that tasks do not need to be long to increase fatigue if they feature for example complex mental task requirements, e.g., high risk for attention failures. Concerning physical work factors, particularly noise, distractions, or temperature play a role (Fan and Smith, 2017). Concerning (2) *process factors* in the course of task achievement, particularly opportunities for recovery or recuperation are essential (Åhsberg, 2000). Pauses in or between tasks enable withdrawal from mentally demanding stressors, reduce activation, restore energy, and allow temporary recovery from work-related fatigue, as also discussed in section Status quo (Geurts and Sonnentag, 2006). Among (3) *person-related factors* subjective efforts of mental exertion are important. Subjectively, complex and intense work leads to experiences of becoming fatigued or mentally exhausted with the key experience of lack of energy. Perceived limitations of task performance can include less accuracy, less focus, increased distractiveness, disengagement or attention shifts (as discussed in section Theories and Models of Prolonged Cognitive Activity). This can lead to higher risks of fatigue-related errors or incidents (Dawson et al., 2012). Since cognitive resources are depleted, professionals seek to compensate through subjective mental effort. Further person-related influences have been attributed to sleep and circadian system factors (Dawson et al., 2012).

With the aforementioned theories, models and frameworks in mind, it becomes clearer how the manner in which MDMs are set-up (with prolonged, consecutive and interactive DM, high caseload and no short breaks for food, water or respite), may be counterproductive by unnecessarily exposing core members to cognitive-behavioral pitfalls (section Cognitive-Behavioral Pitfalls Associated with Time-on-Task), and with that to an increased risk of DM failure. Wide implications for healthcare and patient safety exist since, for example, the excessive night work and working hours are globally recognized as a phenomenon that affects health professionals’ performance and are a leading contributor to medical error and injury (Dawson and Reid, 1997; World Health Organization [WHO], 2009). However, while the European Working Time Directive (2013/88/EC; The European Parliament and Council of the European Union, 2003) protects and restricts excessive working hours with the rest of at least 11 h in a 24 h period, potential detriments in performance due to time-on-task reported in this literature review are not adequately addressed with relevant recommendations. The evidence-based models presented here warrant that not only the number of hours worked in a 24 h period, but also the number of consecutive hours, the type, intensity and complexity of a task that a healthcare professional engages in without adequate opportunities for recovery are given more focus and wider recognition.

## Cognitive-Behavioral Pitfalls Associated with Time-on-Task (the How)

**FIGURE 4:**
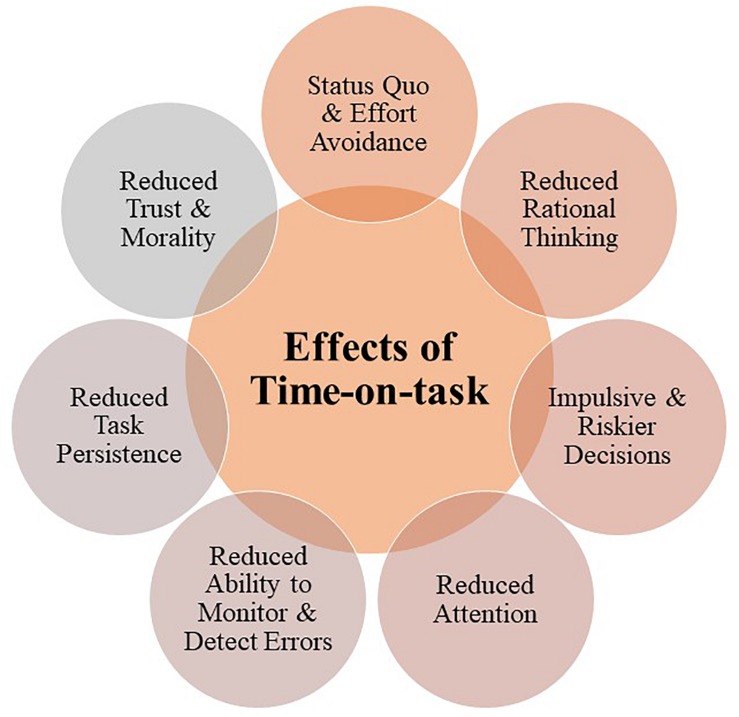
Diagram depicting cognitive-behavioral pitfalls associated with prolonged periods of cognitive activity.

While understanding the underlying mechanisms by which prolonged time-on-task affects task performance is important, identifying the associated cognitive-behavioral pitfalls is equally critical for understanding the potential quality and safety implications for MDMs and designing appropriate management strategies. This is of significance because decrease in the availability of cognitive resources needed for effective DM leads to an array of cognitive-behavioral pitfalls manifested in higher reliance on heuristics, less analytical style of information processing, and simplified decisions (see Figure 4).

### Status Quo

The decision avoidance or status quo have been shown to be a common DM strategy during periods of prolonged time-on-task, because it is less cognitively demanding and energy taxing not to decide than to actively engage in making one (Danziger et al., 2012; Eidelma and Crandall, 2012; Wagner, 2013).

### Rational Thinking

Reduced rational thinking and opting for an immediate but smaller (as opposed to larger but delayed) rewards has also been reported as a result of time-on-task (Wagner, 2013). People were found to perform worse at logic and reasoning, relying more on the “general picture” and jumping to conclusions as opposed to focusing on detail or elaborate, critical processing of information (Webster et al., 1996; Van der Linden et al., 2003a; Van der Linden and Eling, 2006).

### Impulsive Decisions and Poorer Choice Quality

Prolonged time-on-task has been found to lead to more impulsive and riskier decisions, independent of personal experience (Avrahami and Kareev, 2010), personality traits and tendencies at self-control (Freeman and Muraven, 2010). This was evident after controlling for negative mood and stress, variables known to affect DM and risk taking. Research suggests that self-control depletion mediates this relationship since both mood and stress exhaust self-control strength (Bruyneel et al., 2008; Freeman and Muraven, 2010). For instance, after only 10 min of suppressing all internal emotions, and external reactions, cognitive task performance was poorer in comparison to participants who were not required to suppress emotion (Inzlicht and Gutsell, 2007). A study by Mullette-Gillman et al. (2015) showed that after performing a cognitively demanding task lasting 60 min, participants displayed more variable risk attitudes and poorer choice quality on the subsequent economic DM task, indicating lack of consistency in repeating one’s choice given the same options (Mullette-Gillman et al., 2015). However, while the performance on the economic DM task was affected by the preceding cognitively demanding continuous performance task, there was no decline in subsequent task performance despite a significant increase in subjective reports of fatigue in comparison to a control group (who watched a movie and were not exposed to cognitively demanding task; Mullette-Gillman et al., 2015).

### Decrease in Attentional Capacity and Motivation

Decrease in attentional capacity and motivation, including easy distractibility, absentmindedness, reduced ability to effectively evaluate choices and prolonged planning have also been reported as a result of time-on-task (Muraven et al., 1998; Van der Linden et al., 2003b; Boksem et al., 2006; Weissman et al., 2006; Vohs, 2008; Tanaka et al., 2014). For instance, Tanaka et al. (2014) have shown how tasks that require high workload and continuous attention lead to a gradual decrease in attentional capacity, sleepiness, as well as functional changes in the prefrontal cortex associated with promoting task relevant processing. Studies in aviation showed that after long periods of time in flight simulators, pilots experienced reduced attentional capacity and were more easily distracted by noncritical signals, while being less able to detect critical signals (Neri et al., 1992; Neri et al., 2002; Caldwell et al., 2009). In simulated driving studies, errors became more frequent during 3 h of continuous driving, including running off the road and increased speed variations (Campagne et al., 2004).

### Reduced Ability to Monitor and Detect Errors

The ability to monitor and detect errors, which allow available information to be used effectively to plan, prepare and execute an appropriate goal-directed action, were also found to be compromised by time-on-task. Early in task performance (<30 min), participants slow down following an error in order to utilize information from previous tasks to strategically adapt responses and prevent subsequent errors. During the prolonged tasks (>60 min), however, participants try to maintain performance speed by sacrificing accuracy; a phenomenon known as a speed-accuracy trade-off (Scheffers et al., 1999; Lorist et al., 2000, 2005; Van der Linden et al., 2003a; Healy et al., 2004; Boksem et al., 2006; Lorist, 2008; Kato et al., 2009).

### Reduced Task Persistence

Reduced task persistence (Muraven et al., 1998; Vohs, 2008) increased procrastination and impaired performance were reported with previous acts of DM (Vohs, 2008). For instance, Van der Linden et al. (2003b) have shown that time-on-task leads to prolonged planning, as subsequent tasks take longer to complete when cognitive resources are depleted. Aversion toward continuation of the task, reduced commitment and boredom were also reported which, it has been argued, arise as individuals struggled to maintain attention (Sawin and Scerbo, 1995; Boksem et al., 2005; Pattyn et al., 2008).

### Reduced Trust and Ethics

Trust between group members, moral awareness and ethical behavior were also found to be affected during prolonged tasks. For instance, Ainsworth et al. (2014) found in experiments on economic DM that depletion in self-control consistently leads to decreases in behavioral trust in the group. Decrease in moral awareness and increase in dishonesty and unethical behavior (Mead et al., 2009; Barnes et al., 2011; Gino et al., 2011; Yam et al., 2014), hostility and deviance have been documented to increase (Christian and Ellis, 2011).

### Leadership

Leaders (e.g., MDT chairperson) were found to be particularly susceptible to the effects of time-on-task during prolonged cognitive activity. Leadership was found to initially motivate one’s self-control to perform effectively for the group, and leaders were found to employ a specific strategy to deal with depletion by selecting relevant tasks. However, the continued exertion at DM and leading the group takes heavy toll and results in a bigger cognitive impact on their performance (DeWall et al., 2011; Ent et al., 2012).

### What Does This Evidence Mean in Relation to Multidisciplinary Oncology Teams?

By the mechanisms set out above, detriments in performance as a result of time-on-task can inadvertently lead to human error, which is particularly significant for healthcare teams where sustained attention and ability to critically evaluate information are essential for effective formulation of decisions (Christian and Ellis, 2011). During MDMs members engage in a prolonged and effortful cognitive activity that requires DM to be conducted in a sequential and interactive manner for a prolonged period (ranging approximately from 60 to 320 min, which varies for different teams; Cancer Research UK, 2017). This activity exhausts cognitive resources, resulting in depletion of self-regulatory and executive functions that may affect the quality of DM (see Figure 3 for a graphical representation of time-on-task effects and Figure 4 for the associated cognitive-behavioral pitfalls). In what follows, we provide an outline of strategies for preserving the cognitive load during MDMs.

## Cognitive-Behavioral Strategies as Countermeasures of Time-on-Task Effects (the What)

**FIGURE 5:**
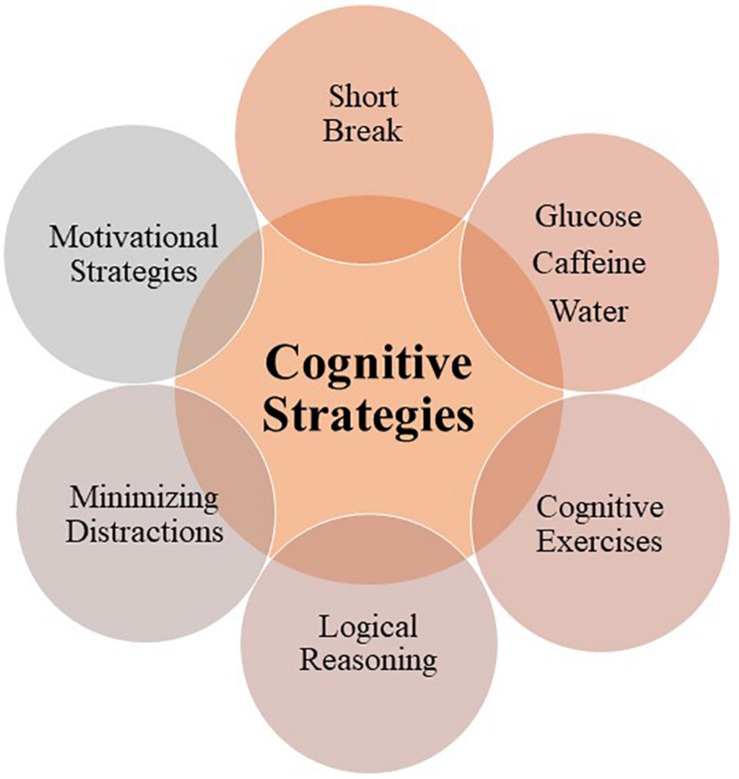
Diagram depicting evidence-based cognitive strategies for mitigating time-on-task effects (in line with the evidence presented in Table 1).

Pursuant to the fact that prolonged cognitive activity can lead to sub-optimal performance and even human error, there is evidence to suggest that implementation of cognitive-behavioral strategies may help restore and enhance performance. *Cognitive strategies* refer to countermeasures or methods that people can use to optimize their cognitive performance by reducing the information processing load, thereby optimizing use of available cognitive resources and mitigating risk of cognitive-behavioral pitfalls, thus reducing negative effects of time-on-task (Halford et al., 2005; Figure 5). Below we provide an overview of such strategies, and in Table 1, we list the associated interventional studies.

### Short Breaks

A *short break* (5–10 min) with food intake, light physical exercise or mindfulness have been found effective in field studies with flight crews, judges and data entry workers for instance, as well as in experimental settings using a dual-task paradigm (i.e., to induce depletion, participants complete an initial self-control task, after which together with the control group they are tested on a second self-control task). Overall, the studies showed a significant improvement in (a) speed without loss of productivity, (b) accuracy and attention and (c) combating biases such as the status quo (something that is particularly appealing for cancer MDMs due to limited time and resources). Physiological measures and unintended sleep episodes were also improved because of a rest break (Neri et al., 2002; Tomporowski, 2003; Galinsky et al., 2007; Tyler and Burns, 2008; Danziger et al., 2012; Friese et al., 2012; Kouchaki and Smith, 2014).

Occupational health and safety sciences also recommend temporary withdrawal from the work itself as well from the tasks that need sustained attention; this includes short-breaks or micropauses, napping as well as establishing shift-schedules that account for circadian rhythms and maintain sleep hygiene (Caldwell et al., 2019). Change of postures or short physical activities can improve alertness and performance briefly (Caldwell et al., 2019). Task design factors are essential that allow for autonomy and discretion to take breaks. If professionals have the autonomy, to have influence on their task load (i.e., job control) and experience psychosocial safety, risks for fatigue are mitigated (Grandjean, 1979). Opportunities for shifting focus away from prolonged tasks enhance recovery experiences, facilitate goal setting and restore energy with the potential reduction in cognitive load (Gander et al., 2011; Herlambang et al., 2019).

### Glucose

*Glucose* was found to enhance performance during prolonged time-on-task, under the hypothesis that glucose, as brain fuel, is required for effortful cognitive processing that relies on increased neural energy mobilization (Donohoe and Benton, 1999; Fairclough and Houston, 2004; Galliot and Baumeister, 2007; McMahon and Scheel, 2010). Correspondingly, studies have shown that blood glucose is sensitive to prolonged cognitive exertion and time-on-task. For instance, in double-blind, placebo controlled, cross-over studies, ingesting sugary drinks led to better performance on numerous self-regulatory tasks: the improvements were evident in working memory, productivity, attention, speed and accuracy, as well as in subjective reports of fatigue, including increased reported alertness and calmness, and reduced reliance on heuristic based automatic reasoning (Kennedy and Scholey, 2000; Scholey et al., 2001; Reay et al., 2006; Masicampo and Baumeister, 2008; McMahon and Scheel, 2010; Owen et al., 2012). Glucose was also found to lead to better self-control for future gain with participants more frequently opting for larger but delayed reward as opposed to a smaller but sooner option (Wang and Dvorak, 2010). However, the literature appears divided on how exactly glucose affects performance i.e., whether it is motivationally or metabolically driven (i.e., the strength versus the process models; (Inzlicht et al., 2014; Carter et al., 2015) with some studies showing no significant effects (Molden et al., 2012; Sanders et al., 2012). It appears that *individual differences* and *task complexity* are important mediators in the rate of glucose metabolization and availability; hence glucose intake may not always show noticeable change in performance (Kennedy and Scholey, 2000; Scholey et al., 2001; Fairclough and Houston, 2004; Owen et al., 2012).

Moreover, Scholey et al. (2001) showed that task complexity affected blood glucose expenditure, i.e., tasks that are cognitively demanding led to a significantly higher reduction in blood glucose in comparison to tasks with minimal cognitive demand. Similarly, Kennedy and Scholey (2000) showed that the tasks perceived as requiring the highest level of mental demand demonstrated the strongest effect of glucose intake. Hence, being able to organize cases in cancer meetings according to their complexity may be beneficial in effectively managing the caseload in order to preserve teams’ cognitive load at an acceptable level.

### Glucose, Caffeine, and Hydration

A combination of *glucose and caffeine*, a mild stimulant (Nehlig, 2010), has also been used as an intervention strategy during prolonged tasks. Studies have shown decrease in subjective feelings of fatigue, as well as improved accuracy on the highly demanding task with a combined glucose/caffeine drink, while on the less difficult tasks, caffeine alone had an effect (Kennedy and Scholey, 2004). In a double-blind, placebo-controlled, cross-ever study, a low does of caffeine (3 mg/kg body weight) led to shorter reaction times and lower error rates in comparison to the placebo group. Physiological response was also observed, i.e., amplitude of the event-related negative potentials, whose key functions are thought to be monitoring of performance, error detection and signaling, was increased within 100 ms following the erroneous response. However, subjective feeling of fatigue did not differ between those that received caffeine and the placebo (Tieges et al., 2004). Nonetheless, high doses caffeine can lead to anxiety, nervousness and jitteriness (Nehlig, 2010). *Staying hydrated* alone may ensure optimal cognitive performance as dehydration can negatively affect short-term memory, perception, attentional vigilance and executive control (Cian et al., 2001; Tomporowski, 2003; Baker et al., 2007; Kempton et al., 2011).

### Motivational Strategies

Various *motivational strategies* have been used as immediate rewards to boost performance without necessarily increasing the metabolic energy level. This is in line with the process model of self-control where glucose is understood to have motivational as opposed to the metabolic effect on cognition. For example, rinsing mouth with glucose, anticipating a small bag of sweets, and monetary reward were found to improve various aspects of performance including persistence, accuracy, speed and information processing (Tice et al., 2007; Krebs et al., 2010; Molden et al., 2012; Sanders et al., 2012; Goto and Kusumi, 2013; Hagger and Chatzisarantis, 2013). In addition, priming participants to feel positive was also found effective, such as for example, asking them to watch a short comedy video prior to the task, encouraging a positive belief about the outcome, as well as using positive altruistic motivation where participants are told that task completion will help a vulnerable population group (Muraven and Slessareva, 2003).

However, *individual differences* exist with one study reporting that the optimism prime led to improvements in task persistence only in those participants who are high in that trait (Yvo et al., 2014). What is more, participants’ levels of fatigue were found to be an important mediator, hence the positive effect of motivation is effective when depletion is mild (i.e., after two initial self-control tasks), but diminishes when extensive (i.e., after four initial self-control tasks; Vohs et al., 2012).

Occupational health and safety sciences also point to motivational aspects. Use of self-directed rewards to foster motivation to recover cognitive resources and to increase willingness to complete tasks are an essential factor in successful achievements of tasks. For instance, use of rewards increases willingness to sustain performance while pursuing mentally demanding, exhausting task goals and helps to restore performance to pre-fatigue levels (Horrey et al., 2011). Yet, it is not investigated how in real-world occupational settings effective processing of information is achieved while being fatigued (Horrey et al., 2011). Nonetheless, individual characteristics such as competence, confidence and self-efficacy, have been reported to successfully dealing with mental fatigue in the workplace (Reiner, 2013).

### Cognitive Exercises

Various *cognitive exercises* were found to attenuate fatigue, too. For instance, when people were encouraged to self-monitor their task performance against a set standard, the depletion effect was eliminated. The researchers introduced a clock, providing an accurate moment-to-moment feedback to participants about the time they had spent on the task, in order to stimulate comparison between their allocation to the persistence task and their standard for such activities. Individual differences were also noted with those low on self-monitoring showing the biggest improvement in performance (Wan and Sternthal, 2008). Another strategy used in previous studies was mental imagery of a restorative activity, i.e., taking the perspective of a person who had already performed a restorative act. However, this effect was only evident when the person used in mental imagery was similar to the participant, i.e., they are both members of the same group, and have a sense of merged group identity (Egan et al., 2012).

### Logical Reasoning

Daily practice of *logical reasoning* using paper and pen workbook or a smart phone application (based on Stroop-task paradigm) can improve self-control, persistence, and speed during prolonged tasks for up to 4 weeks after training (Bertrams and Schmeichel, 2013; Cranwell et al., 2014). This reflects evidence that clinicians engaged in reflective, critical reanalysis of case findings showed improved diagnostic accuracy and reduced reliance on availability and other cognitive biases that occur as a result of recent experiences with similar cases (Mamede et al., 2010b). Arguments have been made that in order to mitigate diagnostic errors and ensure safety, training should be available to healthcare providers in clinical reasoning skills that help increase awareness of cognitive errors and pitfalls in DM (aka cognitive debiasing; Croskerry et al., 2013, 2014). This is also in line with findings from interactive DM research, which show that critical thinking abilities, including adequate evaluation of negative consequences of alternative solutions, problem analysis and establishment of solution criteria are the strongest predictors of effective group DM (Orlitzky and Hirokawa, 2001).

### Minimizing Distractions

Non-interventional studies such as Speier et al. (2007) have found that interruptions lowered DM performance on complex tasks. Increased frequency of interruptions exacerbated the effect, leading to decreased decision accuracy and increased decision time, while the content of interruptions i.e., those containing information that is dissimilar to the primary task, resulted in longer decision time. Working memory was also found to be negatively affected by interferences (Persson et al., 2006). On the other hand, minimizing distractions and taking time for deliberate consideration may be useful strategies since evidence shows that both improve diagnostic accuracy on complex problems in medical experts (Mamede et al., 2010a). This is also important because time pressure is known to hinder performance by encouraging reliance on heuristic processing (Goodie and Crooks, 2004).

### Potential Practical Solutions for Multidisciplinary Oncology Teams

The cost-effective, evidence-based cognitive strategies (Figure 5 and Table 1) discussed in this review could be profitably tested within cancer MDMs since they have a potential to drive clinical improvement. Here we provide some examples as potential practical solutions for MDMs.

**A short break** with refreshments could be beneficial for the team not only as energy restoring, but also as a motivational strategy (see Soukup et al., 2019a for an example of how short break could be introduced to MDMs to improve DM). As such, it could help with overcoming status quo bias and reliance on heuristic-based automatic reasoning, as well as improving working memory, attention, accuracy, persistence, subjective feelings of fatigue, alertness and calmness, moral awareness, and speed without the loss of productivity.

**Encouraging positive mood** with, for instance, pleasant images on the slides during a break, as well as positive encouragement about the general usefulness of MDMs for patient care (e.g., using motivational posters in MDT rooms) are further motivational strategies that can be used in conjunction with other interventions. A short, encouraging introduction by the chair at the MDM’s start may enhance team coherence, motivation and optimism (i.e., similar to preoperative surgical briefings; Mathieu et al., 2000; Lingard et al., 2011).

**Minimizing distractions** and allowing sufficient time for deliberate consideration of each individual case could be effective too; **MDM chair-person** could have a particularly important role in this respect. Having a meeting chair may help by not only minimizing distractions and keeping discussions focused, but also by effectively coordinating the time, workload and complexity of cases to ensure deliberate consideration is appropriately taken within the allocated time frame.

Having a validated tool that captures the **complexity of cases** and helps in streamlining workload, as well as in prioritizing and organizing patient-discussions according to their complexity (e.g., low, moderate, high, see the MeDiC instrument for cancer MDTs, Soukup et al., 2019b) may also prove useful in effectively managing teams’ cognitive load since task complexity was found to increase glucose expenditure (Kennedy and Scholey, 2000). For example, complex cases could be discussed right at the beginning of the meeting (followed by simpler cases) and straight after the break. Teams could also split their weekly MDMs into the complex and straightforward ones with the former consisting of a large and clinically diverse team with one or two short breaks depending on workload, and the latter conducted within a potentially smaller team (Soukup et al., 2019b).

**Regular training and development** sessions could be introduced to provide an opportunity for cancer MDM members (a) to learn and become aware of the pitfalls associated with prolonged cognitive activities, (b) to learn about cognitive strategies and how to use and implement them, and (c) to practice cognitive control and logical reasoning skills. For instance, MDM members could be trained to use mental imagery to help restore self-control when feeling fatigued, and to self-monitor their performance against a set standard (on individual or team level).

Lastly, to support cognitive-behavioral strategies at work, **application of technology** has been advocated since it can provide solutions to mitigate the consequences of fatigue (Dawson et al., 2017; Caldwell et al., 2019). Specific to clinical work, technological advances promise various benefits beyond mere measurement of fatigue such as real-time assessments as well as customization to specific users (i.e., specific to individual needs and states, or creation of individual profiles; Reiner and Krupinski, 2012). Traditionally, technological countermeasures have been suggested that intervene in fatigue-prone decisions or detect potential hazards associated with fatigue (i.e., warnings, automation). Yet, challenges of acceptance, as well as user operability and privacy of data remain (de Bloom et al., 2015; Dawson et al., 2017). In medicine, different technological solutions have been proposed (Dawson et al., 2011), e.g., speech recognition detects various acoustic properties (that are associated with stress measures) with the potential to monitor in real-time, person-specific inflection points in verbal behaviors that indicate increased fatigue (Reiner and Krupinski, 2012). How such technology could be applied and successfully utilized in cancer MDMs is yet to be explored.

## Discussion

The aim of this review was to bring current understanding of prolonged, repeated DM and its consequences of cognitive depletion and detriments in performance into the context of cancer MDMs. Our objective was to address why (section Theories and Models), and how (section Cognitive-Behavioral Pitfalls) prolonged time-on-task affects performance, and what strategies exist to counterbalance negative effects (section Cognitive-Behavioral Strategies as Countermeasures), while proposing practical solutions for improvement.

Cancer MDMs represent a rather unique/distinct part of the cancer care pathway, which with its multifactorial nature (i.e., multiple professionals, treatment options, and patient individual differences, preferences and circumstances; see Figure 2 for a graphical representation of this point). significantly amplifies the complexity of DM. The evidence reviewed here suggests that being engaged in this process on a consecutive basis for a prolonged period of time is likely to present an additional cognitive burden to the MDT members. As such, DM in MDMs relies on effortful information processing, which is exhaustive in short term and its consecutive use leads to depletion, performance detriments over time-on-task and increased risks of cognitive-behavioral pitfalls (e.g., fast impulsive decisions, speed-accuracy trade-offs, reduced attention, motivation, ability to monitor/detect errors, trust and moral awareness) (see Figure 4).

Several theories have been put forward to explain detriments in performance as a result of prolonged cognitive activity. However, due to the complex nature of cancer MDMs, it is arguable that a different approach is needed to adequately explain the phenomenon. We therefore propose a pragmatic framework of repeated DM that brings together current models showing how they complement one another and how they are not necessarily opposing. In exceptional circumstances of some cancer MDMs where 5 h is spent on a task, interaction effects are plausible between what the current models propose, i.e., fatigue, motivational, and attentional factors. We argue that in such complex clinical settings, the performance detriments may be a result of the cognitive control required for effective DM being stretched beyond its limits, therefore leading to shifts in motivation and attention from “*have to*” tasks to “*want to*” tasks (the process model), experience of fatigue (the strength model), and to responses geared more strongly toward immediate bio-energetic high benefits and low costs (the cost-benefit model). Since MDMs are part of a complex organizational infrastructure, it is arguable that each of the three models (i.e., the motivation and attention, fatigue, and bio-energetic) carries different predictive values for different teams depending on their circumstances and characteristics; such as for example duration of the meeting, time of day, tasks undertaken prior to or after the meeting (e.g., some MDT members come from the operating theater or clinics into the meeting), number of breaks undertaken prior to the MDM (if any), individual experience of fatigue, perceived team climate and job satisfaction. Hence no single model on its own provides a comprehensive picture; arguably, they collectively contribute to understanding detriments in performance occurring within a real-world setting of cancer MDMs.

A range of cognitive and behavioral strategies from food and break to rewards and mental exercises have been explored in previous research and found effective as countermeasures of time-on-task effects (see Figure 5). These could be profitably explored for feasibility and acceptability as potential improvement solutions for cancer MDMs (as described in section Potential Practical Solutions for Multidisciplinary Oncology Teams); in particular, since changes to meeting duration and number of patients for MDMs are often not feasible in clinical practice. The MDTs are given strict timelines to make a diagnosis of cancer and then treat the cancer, which means that the team is required to discuss all the patients on the list they are provided. Any changes to the meeting duration (since this would impact which patients get to be discussed with implications for clinical outcomes) would therefore need to be mandated by either policymakers or at an organizational level with careful thought as to how workload can be streamlined so that it does not affect clinical outcomes. This is arguably a catch-22: while increasing the duration of meetings would take resources away from other areas of healthcare, reducing them would further increase the intensity of meetings. Hence, simple and cost-effective cognitive and behavioral strategies (e.g., short break, food; see Figure 5 and Table 1) presented in this review serve as potential solutions to alleviate detriments in performance in ongoing challenging circumstances. Such strategies tend to be easier to adopt and embed into culture than those that are complex and expensive. Nonetheless, such efforts should be followed up with a longer-term imperative of reviewing capacity within cancer services and how best to plan workforce development and service delivery models to achieve population coverage whilst maintaining safety and quality of care.

### Limitations

This extensive review is an attempt to translate and apply current scientific understanding on the topic of time-on-task effects to cancer MDMs. MDT specialists are facing a real workload issue due to population trends, cancer incidence and financial pressures on healthcare. Building on existing behavioral science insights, our review attempts to start the conversation and offers a framework through which the work of these specialist teams can be better understood and planned – something that to our knowledge has never been attempted before. However, this is not a systematic review or meta-analysis and results must be interpreted with caution. What is more, we have not linked the findings we present here to clinical, patient-related outcomes in a statistical manner (only conceptually), and as a result, the safety implications of our review remain exploratory and are not yet equated to clinical outcomes.

We also acknowledge that the challenges in the field of time-on-task effects exist, and we encourage the readers to review recent meta-analyses (Hagger et al., 2010; Carter et al., 2015; Tuk et al., 2015) covered in section Theories and Models of Prolonged Time-on-Task where varying effect sizes in laboratory studies have been reported; as well as the more recent review on the conceptual crisis in the field that calls for more research involving larger samples and preregistration (Lurquin and Miyake, 2017).

We also acknowledge that occupational health evidence concerning effective approaches to reduce mental fatigue spans across cognitive-behavioral strategies proposed in this review. Fatigue-proofing strategies have been proposed that are adaptive to local work systems and operational circumstances and that foster resilience of a system to mitigate the likelihood of adverse consequences of fatigue-prone tasks (Reiner, 2013; Steege and Dykstra, 2016; Steege et al., 2017). Hereby, a combination of fatigue-reduction strategies that address contextual factors of the work environment (i.e., task characteristics, working time regimes) as well as individual behavior-based approaches (e.g., self-monitoring; Dawson et al., 2017) is suggested. In practice, broader approaches embedded into the wider organizational system or formal safety management systems with systematic identification and evaluation of fatigue-proofing interventions need to be developed (Reiner, 2013; Steege and Dykstra, 2016; Steege et al., 2017; Caldwell et al., 2019). As an example, in the transport sector multi-dimension fatigue risk-management systems (FRMS) have been advocated on macro-levels (i.e., organizations, industry, regulatory bodies) to shift the focus of responsibility from individuals to cultural and regulatory levels (Techera et al., 2016; Dawson et al., 2017). However, in clinical work settings, such systems are rarely implemented (Reiner, 2013).

### Future Research

Establishing evidence-based cognitive-behavioral strategies (Figure 5 and Table 1) for cancer teams emerges arguably as a priority for improving this particularly important part of the cancer care pathway. While there are no studies to date, to our knowledge, that have directly assessed effects of time-on-task and relevant interventions within the context of cancer MDMs, it is important to consolidate the existing body of knowledge in order to make improvements. Equally, there is a genuine lack of field research exploring the phenomena of time-on-task with most findings to date coming from laboratory studies apart from Soukup et al. (2019a) for breast cancer MDMs (*N* = 1335 patient-discussions), Lamb et al. (2013a) for urological MDMs (*N* = 1421 patient-discussions), Danziger et al. (2012) for courtrooms (*N* = 1221 judicial decisions), and Harewood et al. (2009) for endoscopy procedures (*N* = 400 procedures). Further research is therefore essential in order to (a) validate current theories in an applied setting and on large samples, and help provide more stable estimates of effect sizes, (b) explore them in relation to task complexity, (c) gain specific scientific understanding of the time-on-task effects in clinical contexts and (d) how such specialist teams can function better within the existing resourcing envelope, so that team- and cancer-specific strategies can be developed and applied. Such strategies will become performance-critical in the future, in the light of increased cancer rates (Mistry et al., 2011; World Health Organization [WHO], 2014), and financial pressures on health systems (NHS England, 2014; World Health Organization [WHO], 2014), but also in terms of patient safety and team working (Reader et al., 2006, 2011).

Building understanding of the MDT members subjective experience of fatigue and effort as a result of MDMs should also be explored, although such investigations may need to overcome practical challenges in terms of the feasibility of such psycho-physiological assessment measures (due to a fast paced environment and a potential to impinge on clinical time, hence research on MDMs has been predominantly observational).

### Potential Implications

The evidence to do with detriments in performance that arise because of excessive night work and working hours (or, shift work) is far reaching (World Health Organization [WHO], 2009; The Joint Commission, 2011), reportedly, leading to a 300% increase in preventable adverse events and fatalities (NHS Improvement, 2016). Hence the European Working Time Directive (2013/88/EC; 69) protects healthcare workers’ health and safety, restricting working hours with the rest of at least 11 h in a 24 h period. However, the field studies (i.e., Harewood et al., 2009; Danziger et al., 2012; Lamb et al., 2013a; Soukup et al., 2019a), and the current theories and models (i.e., the strength model of self-control pointing to decision-making fatigue, and the process and cost-benefit models highlighting the attentional and motivational factors), all point to the potential detriments in clinical performance as a result of the intensity (time-on-task) and complexity of the workload. In contrast to the management of excessive night work and working hours, this is something that is not adequately addressed or acknowledged – such that relevant recommendations and rest breaks, for example, are not formally enforced. It is understood however that the intense episodes of workload in healthcare are on the increase (Mistry et al., 2011; NHS England, 2014; World Health Organization [WHO], 2014), even more so as the clinical teams are trying to maximize productivity in the face of severe workforce shortages (NHS Improvement, 2016) and financial pressures (NHS England, 2014; World Health Organization [WHO], 2014). Hence it is not only the number of hours worked in a 24 h period, but also the number of consecutive hours including the type and the complexity of the task (i.e., how cognitively demanding the task is) a healthcare professional engages in without adequate break for respite, that require more research and regulatory focus and recognition. As one MDT member put it: “Sometimes we discuss up to 70 patients. This is after a whole day of clinics and we don’t finish until gone 19.00. Would you want to be number 70?” (Cancer Research UK, 2017).

## Conclusion

Scientifically, there is a strong case to pursue the effort of unpacking different facets of time-on-task effects that the strength, process and cost-benefit models with accompanying (although limited) field evidence highlight. Arguably, MDMs represent an invaluable segment of the cancer care pathway with the emerging evidence showing that when it comes to DM, groups are largely more rational than individuals. Due to increasingly complex and specialized care, they are also an integral part of service delivery across healthcare systems with an effective team work helping promote patient safety. They are in many ways unique: at no other point of cancer care pathway (or even other settings) do we see such simultaneous factor interactions when it comes to DM (Figure 2). In MDMs, an array of interdisciplinary professionals are required to work together, consider multiple treatment options and diverse patient circumstances, and reach a consensus for treatment recommendations for each patient, repeatedly, and under uncertainty about the consequences of chosen options. Given recent scientific advances from psychology, neuroscience and behavioral economics to organizational and consumer behavior in the understanding of the effects of time-on-task on performance, the time appears ripe for research efforts to consolidate and apply the existing knowledge to cancer MDMs – for improved wellbeing of health care providers and patient care.

## Author Contributions

TS, NS, and BL contributed conception and design of the review. TS organized the literature. TS wrote the first draft of the manuscript. BL, JG, MW, and NS wrote sections of the manuscript. All authors contributed to manuscript revision, read and approved the submitted version.

## Conflict of Interest Statement

NS is the director of London Safety and Training Solutions Ltd., which delivers human factors, patient safety and quality improvement advisory and training services to hospitals and training programs globally. JG is a Director of Green Cross Medical Ltd., that developed MDT FIT for use by National Health Service Cancer Teams in the United Kingdom. The remaining authors declare that the research was conducted in the absence of any commercial or financial relationships that could be construed as a potential conflict of interest. The reviewer GR declared a shared affiliation, with no collaboration, with one of the authors, BL, to the handling Editor at the time of review.

## Appendix Key Concepts

• **Executive function/cognitive control** is an umbrella term for a set of higher-order processes required to purposefully control, oversee and regulate one’s various cognitive resources while working toward a specific goal (*aka* top-down processing). Executive functions include attentional control, inhibitory control, working memory, cognitive flexibility, reasoning, problem solving, planning and DM. In the brain, executive function is thought to be mediated by the areas of the frontal lobe.
• **Self-control/self-regulation** is an ability to override one’s initial or predominant response tendencies and align the behavior with internal intentions, goals and standards.
• **Mental fatigue** is a broad term pertaining to a decrease in optimal cognitive and behavioral performance resulting from prolonged periods of cognitive activity that requires sustained mental efficiency.
• **Executive/cognitive depletion** is a decrease in the ability of higher-order processes to purposefully control, oversee and regulate cognitive resources.
• **Self-control or self-regulatory depletion** is a decrease in the ability to override one’s initial or predominant response tendencies, and align behavior with internal intentions, goals and standards.
• **Decision-making fatigue** is a decrease in optimal DM resulting from prolonged periods of repeated DM that requires sustained mental efficiency.
• **The strength model of self-control** postulates that self-control is a limited resource and depletion arises because of this.
• **The process model of self-control** postulates that self-control depletion is a result of shifts in attention and motivation.
• **The cost-benefit model of self-control** postulates that self-control depletion is a result of inefficient evaluation of costs and benefits by responding more strongly to the immediate rewards i.e., high benefit and low cost.

## Abbreviations

DM: decision-Making
MDM: multidisciplinary team meeting
MDT: multidisciplinary team.
